# The eggshell membrane: A potential biomaterial for corneal wound healing

**DOI:** 10.1177/08853282211024040

**Published:** 2021-06-18

**Authors:** Rosemond A Mensah, Seung Bin Jo, Hoon Kim, Sung-Min Park, Kapil D Patel, Kyong J Cho, Michael T Cook, Stewart B Kirton, Victoria Hutter, Laura E Sidney, Decio Alves-Lima, Hungyen Lin, Jung-Hwan Lee, Hae-Won Kim, David YS Chau

**Affiliations:** 1 School of Clinical and Pharmaceutical Sciences, University of Hertfordshire, Hatfield, UK; 2 Eastman Dental Institute, University College London, London, UK; 3 Institute of Tissue Regeneration Engineering, Dankook University, Cheonan, Republic of Korea; 4 Department of Ophthalmology, Dankook University College of Medicine, Dankook University, Cheonan, Republic of Korea; 5 Academic Ophthalmology, Division of Clinical Neuroscience, School of Medicine, University of Nottingham, Nottingham, UK; 6 Department of Engineering, Lancaster University, Lancaster, UK; 7 UCL Eastman-Korea Dental Medicine Innovation Centre, Dankook University, Cheonan, Republic of Korea; 8 Department of Nanobiomedical Science and BK21 NBM Global Research Centre for Regenerative Medicine, Dankook University, Cheonan, Republic of Korea

**Keywords:** ECM, tissue engineering, cell culture, biomimetic, membrane, wound dressing

## Abstract

The eggshell membrane (ESM) is an abundant resource with innate complex structure and composition provided by nature. With at least 60 million tonnes of hen eggs produced globally per annum, utilisation of this waste resource is highly attractive in positively impacting sustainability worldwide. Given the morphology and mechanical properties of this membrane, it has great potential as a biomaterials for wound dressing. However, to date, no studies have demonstrated nor reported this application. As such, the objective of this investigation was to identify and optimise a reproducible extraction protocol of the ESM and to assess the physical, chemical, mechanical and biological properties of the substrate with a view to use as a wound dressing. ESM samples were isolated by either manual peeling (ESM-strip) or via extraction using acetic acid [ESM-A0.5] or ethylenediaminetetraacetic acid, EDTA [ESM-E0.9]. Energy dispersive X-ray spectroscopy (EDS) confirmed that there were no traces of calcium residues from the extraction process. Fourier transform infrared (FTIR) spectroscopy revealed that the extraction method (acetic acid and EDTA) did not alter the chemical structures of the ESM and also clarified the composition of the fibrous proteins of the ESM. Scanning electron microscopy (SEM) analyses revealed a three-layer composite structure of the ESM: an inner layer as continuous, dense and non-fibrous (limiting membrane), a middle layer with a network of fibres (inner shell membrane) and the outer layer (outer shell membrane) of larger fibres. Material properties including optical transparency, porosity, fluid absorption/uptake, thermal stability, mechanical profiling of the ESM samples were performed and demonstrated suitable profiles for translational applications. Biological *in vitro* studies using SV40 immortalised corneal epithelial cells (ihCEC) and corneal mesenchymal stromal cells (C-MSC) demonstrated excellent biocompatibility. Taken together, these results document the development of a novel sustainable biomaterial that may be used for ophthalmic wounds and/or other biomedical therapies.

## Introduction

A key function of the cornea is to maintain a tough, physical and impermeable barrier between the eye and the environment. As such, the cornea is prone to eye injuries such as physical/chemical trauma or severe infections- often leading to irreparable damage to the tissue and potentially blindness.^1–3^ Therefore, it is essential for the cornea structure to be restored rapidly after injury to prevent the permanent loss of vision. Currently the most widely used biological material for cornea repair/regeneration is the amniotic membrane (AM).^2,4–7^ However, limitations associated with the AM includes variation of the thickness, mechanical strength and transparency of the membrane at different parts of the membrane, donor-to-donor variations, use of anti-rejection drugs/therapy, and ethical and (local) regulatory restrictions.^3,5,7–12^ In addition, the cost of serological testing, processing and preserving of the AM is relatively expensive.^5,8–10^

**Figure 1.**
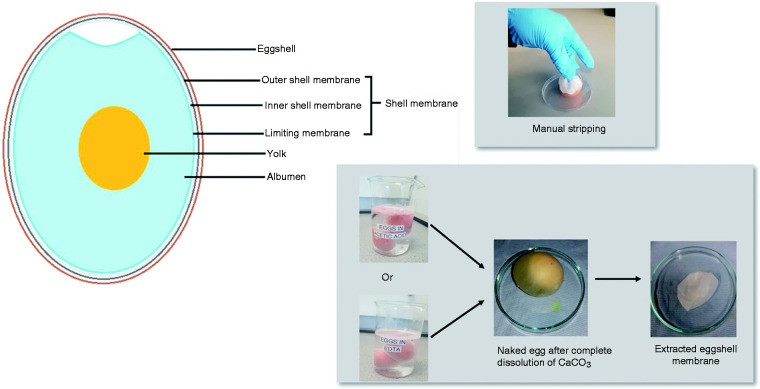
(a) Schematic diagram showing the anatomy of chicken egg; (b) photographic overview of the different protocols used for membrane extraction.

The chicken (*gallus gallus*) eggshell membrane (ESM) is an exceptional biomaterial in nature with its usefulness being underestimated as it is considered to be a waste material. Nonetheless, researchers have made insightful findings whilst investigating this biomaterial because of its unique properties and defined structure.^13–20^ The ESM is a protein-based fibrous tissue that lies in between the mineralized eggshell (ES) and egg white (albumin) (Figure 1). The ESM has a wide content of bioactive components and exceptional biocompatibility/biodegradability properties which has implicated it use as a potential drug delivery system.^13–21^ Its bioadhesive properties has also been investigated as a candidate for a novel oral dosage form.^22^ In its native form, the membrane contains collagens type I, V and X, fibronectin, proteoglycans and glycoproteins^14–20^ and can be seen as three distinct stratified substructures: the outer shell membrane, inner shell membrane and the limiting membrane.^15,21^ The outer ESM is located just under the ES and its fibres range in thickness between 1 and 7 µm. The fibres of the outer shell membrane extend into the mammillary knobs of the shell. In comparison with the outer membrane, the fibres of the inner membrane are smaller in diameter, their thickness ranges from 0.1 to 3 µm and, additionally, the whole membrane layer is generally thinner.^14–16^ Fibres of the inner ESM are observed to be interlaced with the outer membrane. The limiting membrane represents the innermost, very thin, dense structure (non-fibrous) of the ESM, which surrounds the egg white.^14,20–25^ ^Bellairs and Boyde^21^ reported that after staining the eggshell membrane with fluorescein isothiocyanate (FITC), the limiting membrane appears as particles that fill the spaces between the inner membrane fibres, several microns outward from the level at which the inner membrane fibres first appears.

The ability to separate ESM successfully from the ES is a vital procedure for its extensive use as a biomaterial. The extraction of the membrane from the ES has been investigated in several reports: direct manual peeling of membrane from the shell is one of the methods reported to be efficient in preparing ESM despite the fact that the outer membrane reamins strongly bounded to the shell.^2,5,7–11^ Alterntatively, the ES could be exposed to a solvent- essentailly, an acidic treatment could be applied to the ES which acts on its surface by dissolving the calcium carbonate within the shell and loosening the interfacial bonding between outer shell membrane and the ES.^14,18,25^ Interestingly, a recent study has exploited the soluble eggshell protein (SEP) fraction, extracted from raw eggshell membrane, as an possibel enhancement factor to develop modified electrospun nanofibrous scaffolds.^26^

Translational applications of the ESM as a biomaterial can be supported by its chemical composition and physical/mechanical characteristics. However, the uptake of its use as a wound healing agent, in the form of a bioactive dressing for skin applications, has been limited to date.^15,27–29,30^ Despite a very limited summary on the first use of the ESM in ophthalmology in 1899, when Coover successfully used it in a surgical procedure as a bandage for corneal ulcer,^31^ to date, no other research data or studies on the direct use of ESM in ophthlamic application have been reported. Accordingly, with the increase incidents of ocular disease, trauma, tissue replacement and pharmaceutical drug development, the exploitaton of the ESM for corneal wound healing may have been overlooked. As such, the aim of this study was to investigate the development of a novel protocol, using acetic acid and ethylenediaminetetraacetic acid (EDTA), to extract the eggshell membrane and, thereafter, to evaluate their physical, mechanical and biological properties in the context of translational ocular surface therapeutic applications.

## Materials and methods

### Materials

Free-range, brown, chicken eggs (British Blacktail, *gallus galuus*) were purchased from a local supermarket (Waitrose, London, UK). Acetic acid, ≥99% was purchased from Fisher Scientific (Loughborough, Leicester, UK). Ethylenediaminetetraacetic acid (EDTA), hexamethyldisilane, ≥99%, (HMDS), cacodylate buffer, 0.1 M (CAB), 0.25% (v/v) trypsin-EDTA, 100 mM L-glutamine, Antibiotic-Antimycotic (AbAm), M199 medium, Giemsa stain and May-Grunwald stain were obtained from Merck (Poole, Dorset, UK). Glutaraldehyde was supplied by Agar Scientific (Stansted, Essex, UK). Epilife Medium with calcium and Human Keratinocyte Growth Supplement (HKGS) were purchased from Invitrogen Life Technologies (ThermoFisher, Leicester, UK). CellTiter 96® AQueous One Solution Cell Proliferation assay (i.e. MTS assay) and the CytoTox-ONE™ Homogeneous Membrane Integrity assay kits (i.e. LDH assay) were obtained from Promega (Southampton, Hampshire, UK). All other reagents and chemicals were obtained from Merck (Poole, Dorset, UK) unless otherwise stated.

### Membrane extraction

Fresh eggs were washed carefully with DI water before incubating them at room temperature (∼19°C), subermerged, in 0.5 M acetic acid for 44 h (ESM-A0.5) or 0.9 M EDTA for 20 h (ESM-E0.9). A preliminary study was performed to obtain these optimised concentrations and durations (data not shown). After the complete dissolution of the calcium carbonate shell by visual observation, the extracted memebranes were collected and washed in DI thoroughly to remove the albumen and yolk (Figure 1(b)). As a control, the ESM was stripped off manually from the eggshell using tweezers (ESM-strip). All resulting extracted ESM samples were fully immersed in PBS in order to avoid dehydration and stored in a refrigerator (4°C) before use. All experiments were performed with ESM samples that had not been stored for longer than 72 h.

### Membrane characterisation

#### Thickness

The thickness of the extracted membranes were measured by placing the membranes (dabbed “dry” using paper towel to remove excesss water) between two microscopic slides of known thickness. The measurements of the total sandwich were taken to the nearest 0.01 mm using Moore & Wright Outside micrometer (Zoro, Leicester, UK). The thickness of each membrane was measured at six random locations and the average values were reported as to be the membrane thickness.

#### Optical properties

ESM samples were soaked in PBS for 24 h to equilibrate before their transparency characteristics were assessed using two different techniques. In the first case, wet membranes were placed over a standardised waterproof test card and the images of the inner and outer surfaces of each membrane were taken using a 12MP Super Speed Dual Pixel AF sensor camera (OIS, FOV: 77°, Dual Aperture: F1.5 mode/F2.4 mode) on the Samsury Galaxy S9 plus cellphone (protocol adapted from Chau et al.^5^) In contrast, the second assessment technique was based on the measurement of light transmittance through the wet membranes and determined using a T80 UV-VIS spectrophotometer (PG intrusment *Ltd*., Leicester, UK) at a wavelength range from 400 to 1000 nm.

#### Scanning electronic microscopy

The surface morphologies and characteristics of the outer and inner sides of each extracted membranes were obtained using field emission scanning electron microscope, FESEM, (Philips XL30, UK) with operation voltage of 5 kV, spot size 3. Before examination, membranes were fixed in 3% (w/v) glutaradehyde in 0.1 M cacodylate buffer for 24 h at 4°C. The fixed membranes were then dehydrated in a series of graded ethyl alcohol solutions for 2 min each: 1 × 70%, 1 × 90% and 3 × 100%. Thereafter, the membranes were critical-point dried by immersing in HMDS for 2 min. The dried membranes were attached onto adhesive 12 mm carbon tabs (Agar Scientific, Stansted, UK) which were pre-mounted onto 0.5 aluminium spectrum stubs (Agar Scientific, UK) before being sputter-coated with gold/palladium (Polaron E500, Quorum Technology, UK). Morphologies of the membranes were analysed at magnification of ×500 and ×2000. In addition, the elemental composition of the extracted membranes were analysed by FESEM with energy dispersive X-ray (EDX) attachment operating at 15 kV, spot size 5.

#### Fourier-transform infrared spectroscopy

The elements and functional groups of the ESM samples were determined using PerkinElmer Fourier-transform infrared spectroscopy (FTIR) operating in the Attenuated Total Reflectance mode (SensIR Technologies, UK). The samples were scanned in the IR range from 600 to 4000 cm^−1^ and determined at 20°C. The spectromemter was calibrated by taking a background spectrum before analysing the extracted membranes.

#### Porosity

The fluid handling property of the membranes were assessed by determining the porosity using displacement method described by Ahmed and Boateng,^32^ with a slight modification. Membranes were air dried, at room temperature (∼19°C), for 24 h and weighed. The dried samples were then immersed in 5 ml of PBS, at 34°C, for 24 h and weighed after dabbing the surfaces with paper towel. The average thickness (mm) and effective area (mm^2^) of the membranes were used to determine the total pore volume. The membrane porosity (ɛ) was calculated using equation (1) as stated below (*n* = 6)

(1) $$ɛ\left(\%\right)=\frac{\mathrm{Wet}\;\mathrm{weight}-\mathrm{Dry}\;\mathrm{weight}}{\mathrm{Density}\;of\;\mathrm{PBS}\;\times \;\mathrm{total}\;\mathrm{pole}\;\mathrm{volume}}\times 100$$

#### Fluid absorption

The extracted membranes were cut into 2 × 2 cm squares and air dried at room temperature (∼19°C) for 24 h. Membranes were weighed to the nearest 0.1 mg. The fluid absorption (FA) of each membrane was determined by immersing the samples in 5 ml of PBS, at 34°C, for an hour and 24 h. After 1 and 24 h, the membranes were carefully blotted using paper towel to remove excess PBS and weighed. Each measurment was repeated six times. The FA was calculated using equation (2) as stated below

(2) $$FA\left(\%\right)=\frac{\mathrm{Wet}\;\mathrm{weight}-\mathrm{Dry}\;\mathrm{weight}}{\mathrm{Dry}\;\mathrm{weight}}\times 100\;$$

#### Swelling index

The swelling index (SI) of each membrane was performed by immersing the ESM samples into 5 ml of PBS, at 34°C, and any change in the weight of the swollen membrane recorded at 2-min intervals for a total of 10 min before being monitored every 10 min afterwards until 1 h total time. The SI was calculated using equation (3) as stated below (*n* = 6).

(3) $$SI\left(\%\right)=\frac{\mathrm{Swollen}\;\mathrm{weight}-\mathrm{Dry}\;\mathrm{weight}}{\mathrm{Dry}\;\mathrm{weight}}\times 100$$

#### Thermogravitational analysis

The thermal decompositon profile of the extracted membranes was analysed using a 2050 TA thermogravimetric analyser (TGA) (TA instruments, Crawley, UK). The weighed membranes (5–10 mg) were placed into aluminium pans and then deposited within the TGA instrument sample chamber. Each membrane was subjected to a heating profile of 17–150°C, at a rate of 10°C/min, under 20 ml/min nitrogen flow. Weight loss of each membrane was deduced from a standardised TGA analysis protocol.^33^

#### Contact angle measurements

Hydrophilicity of the ESM samples was determined by measuring the contact angles (CA) of both the outer and inner surfaces using the sessile drop method as previously described.^33^ In short, a small droplet of PBS solution (∼2.0 µL) was deposited on the horizontal membrane surface and the contact angle was measured using CAM 200 optical contact angle meter (KSV Instruments Ltd, Finland) at room temperature (∼19°C).

#### Water drying profile

Membrane water drying profile were determined using terahertz (THz) sensing.^34^ Compared to conventional gravimetric analysis, this technique can be performed without physical contact on the sample of interest. The transmission geometry of the technique differs to reflection geometry^35,36^ by allowing water content to be quantified. To ensure full hydration, the membranes were immersed in distilled water for 24 h. Prior to measurement, excess surface water were removed. By monitoring the relative THz intensities, weight of water across each membrane (EW) can be estimated as a function of drying time using Beer-Lambert Law, under the assumption of constant water density and uniformity. In particular, equation (4) was used to convert the measured intensities into estimated weight

(4) $$EW=\frac{A_{\mathrm{membrane}}}{A_{\mathrm{camera}}}\sum_{\mathrm{pixel}=1}^{100}\frac{-{\left(l_{\mathrm{pixe}l}\right)}^{2}\rho \ln\left(\frac{I_{\mathrm{pixel}}}{I_{0,\mathrm{pixel}}}\right)}{\alpha }$$

where l_pixel_ is the pixel size (1.5 mm), *ρ* is the water density at 25°C (1 g/cm^3^), I_pixel_ and I_0,pixel_ are the light intensities for hydrated and dried membrane, respectively, on each pixel and *α* is the absorption coefficient of water at 100 GHz and 25°C (11 mm^−1^). Measurement across 10 × 10 (15 × 15 mm^2^) pixel matrix (A_camera_) is then used for liquid water weight estimation and extrapolated to the entire membrane area (A_membrane_) for a total weight.

#### Texture analysis

##### Compression properties

**Figure 2.**
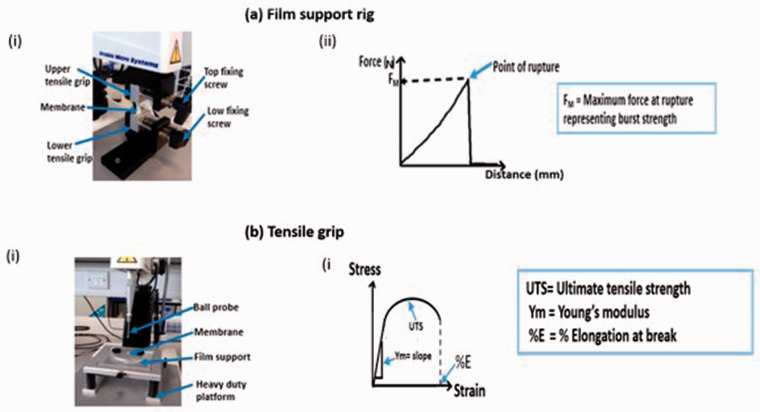
Membrane characterisation using the TA.XT texture analyser (Stable Micro Systems Ltd., Surrey UK) measured and associated adaptions used for specific tests (ai) a film support rig attached to TA.XT instrument to assess the burst strength. The rig is aligned with the ball probe to ensure the probe moves carefully through the test membrane without touching the film support rig. As the probe deflected the test membrane, the forced was increased until membrane rupture was observed. The maximum force representing the burst strength was recorded (aii) Graphical representation of a typical burst strength test generated by the texture analyser film support rig setup (bi) Tensile grip attached to TA.XT instrument measured to measure tensile strength and the extensibility of the samples. Membranes were sandwiched between sheets of sandpaper and clamped securely. Data profiles were recorded when the force equalled the trigger force (bii) Graphical representation of typical stress and strain curve generated by the tensile measurement setup.

The burst strength of the wet membranes was measured by attaching a film support rig to the Texture Analyser TA. XT (Stable Micro Systems Ltd, Surrey UK) (Figure 2). The test membrane (the outer and inner sides of each membrane) was supported between plates which exposed a circular section of the membrane. The test was performed by initially moving the ball probe at a pre-test speed at 2 mm/s. When the probe reached the surface of the membrane and a trigger force of 5 g was obtained, the speed of the probe was changed to 1 mm/s which initiated the actual test protocol. As the probe deflected the test membrane, the forced was increased until the rupture of the membrane was achieved. The maximum force representing the burst strength was recorded and the distance to burst was recorded as the displacement.^37^ All experiments were replicated six times and conducted at room temperature (∼19°C).

##### Tension properties

The ultimate tensile strength, elongation at break and Young’s modulus of the ESM samples were measured using a tensile grip (Figure 2(b)) attached to Texture Analyser TA. XT (Stable Micro Systems Ltd, Surrey UK) with a 5 kg load cell at 10 mm/s. Prior to analyses, samples were cut into a bone shape with height and length as 25 × 10 mm, respectively. Each test membrane was supported by a standard sandpaper to enhance friction and to prevent slipping in between the grips during the analyses before being carefully placed in between the two grips and the sandwich was then screwed tightly together. The extension of test sample caused an increase in force. Data were recorded when the force equalled the trigger force.^25,38,39^ The tensile properties and ultimate tensile strength were obtained from the TA machine and Young’s modulus was calculated using equation (5) as stated below^32^

(5) $$\begin{array}{l} \mathrm{Youn}g\text{'}s\;\mathrm{modulus}\;\left(\mathrm{MPa}\right)\\ =\frac{\mathrm{Slope}}{\mathrm{Membrane}\;\mathrm{thickness}\times \mathrm{speed}\;(mm/\sec)}\times 100\; \end{array}$$

### Biological characterisation

#### In vitro cytotoxicity evaluation

Human tissue for research was obtained from Manchester Eye Bank subject to a Materials Transfer Agreement and stored and disposed according to the tenets of the UK Human Tissue Act. C-MSC were isolated from corneoscleral rims as previously described.^40^ C-MSC were cultured in M199 medium supplemented with 20% (v/v) FBS, 1% (v/v) AbAm and 2 mM l-glutamine. The SV40 Immortalised Corneal Epithelial Cell Line (ihCEC) was cultured using Epilife medium supplemented with 5 ml HKGS and 1% (v/v) AbAm. Routine cell culture involved the use of Corning T-75 flasks (Corming Life Sciences, UK), standard trypsinisation protocol (i.e. 0.25% (v/v) trypsin-EDTA) and placement within a humidified incubator, at 37°C and 5% CO_2_. Sample membranes (i.e. ESM-strip, ESM-A0.5 and ESM-E0.9) were cut into 14 mm (diameter) discs using a circular craft punch. The samples were then placed into Corning Costar™ Ultra-Low Attachment 24-well tissue culture plates, TCP, (Merck, Poole, UK) and sterilised using 1% (v/v) AbAm, in PBS, for 24 h before an additional treament of UV irradiation, for 2 h, in the laminar cell culture flow hood (Thermo Scientific, Germany). Thereafter, 200 µL of complete growth media were added to the each sample in the well and incubated for 30 min to allow for “pre-wetting” of the membrane samples. The ihCEC or C-MSC cells were seeded on the inner sides of the samples (LESM-strip, LESM-A0.5 and LESM-E0.9), outer sides (OESM-strip, OESM-A0.5 and OESM-E0.9) or TCP at a density of 2 × 10^4^ cells/cm^2^ or 10^4^ cells/cm^2^, respectively, in 200 µL of the complete growth media and incubated for one, three or seven days under standard cell culure conditions (5% CO_2_, 37°C). Control groups using TCP, the inner sides or outer sides of samples with media only were also included. Each sample group had six replicate studies.

The metabolic activities of the cells were evalauted using the CellTiter 96 AQueous One Solution Cell Proliferation assay (Promega, Southampthon, UK) according to the manufacturer’s protocol. In brief, after one, three or seven days incubation, 50 µL of the culture media from the wells of three samples in each group were transferred to new 96 well plate and retained for the LDH assay as described below. Following on, 30 µL of the CellTiter One reagent was then added to each well and incubated at 37°C for 3 h. Subsequently, 100 µL of the media were transferred from the wells in each group into a new 96 well plate and absorbance was read at 492 nm using a BioTek micro plate reader (BioTek, Swindon, UK). For each time point, samples with media only controls were used as the background control.

To quantify lactate dehydrogenase (LDH) release from the cells, the CytoTox-One Homogeneous Membrane Integrity assay kit was used. 50 µL of the CytoTox-One reagent were added to the 50 µL culture media (removed as mentioned above) in the 96 well plate and incubated, in the dark/covered in foil, at room temperature (∼19°C) for 10 min. Thereafter, 10 µL of stop solution was added to each well and the sample plate read immediately using the BioTek micro plate reader (525 nm excitation wavelength and 560–590 nm emission wavelength). Samples with media only controls (background control) were subtracted from the corresponding wells to attain the corrected fluorescent reading.

#### Cell attachment and spreading

The attachment and spreading characteristics of the cell on the membranes were evaluated using an adapted version of the protocol previously described by Shafaie et al.^41^ Media from three sample wells from each group (i.e. samples with cells) were aspirated and cells were then mwashed twice with 100 µL of PBS solution. Sequential addition of 100 µL 3.7% (w/v) paraforlaldehyde in PBS solution for 15 min, followed by a PBS wash step, and then 100 µL of 1% (v/v) Triton X-100, for 15 min, were used to fix and permealise the cells at room temperature (∼19°C), respectively. Thereafter, 100 µL of 0.25% May–Grunwald stain, in methanol, were used to treat at room temperature for 15 min. 100 µL of Giemsa stain, in methanol, (1:20 dilution with distilled water) were added to stain the nuclear membranes of the cells. Stains were removed following a 20-min incubation time, at room temperature (∼19°C), before being washed once with distilled water and air dried for 2 h, at room temperature (∼19°C). Cell samples were visualised and imaged using an optical Meiji EMT microscope (Meiji Techno, Somerset, UK) and GX CAM digital camera, and also the FESEM (Philip XL30, UK) with operation voltage of 5 kV, spot size 3 and ×100 magnification.

#### Angiogenic properties

**Figure 3.**
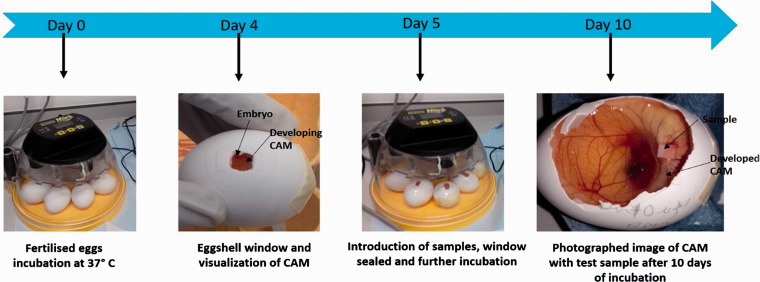
Angiogenesis assessement using the CAM assay. Flowchart summarising the protocol followed when using fertilized eggs, incubated with the ESM samples, to compare blood vessel formation.

The *in ovo* chicken embryo chorioallantoic membrane (CAM) assay (Figure 3) was used to ascetrain the angiogenic potential of the (inner and outer sides) of the extracted membranes using a slightly modified version of the protocol previously described by Chau et al. .^5^ Briefly, fertilised Dekalb White chicken eggs (Henry Stewart & Co *Ltd*, Norfolk, UK) were incubated in a Brinsea Eco incubator for four days at 37°C and 80% relative humidty. On the fourth day, 5 ml of the egg white was taken out using a blunt 18 gauge needle through a hole to decrease the volume space within the egg and result in a lower/detachment of the CAM from the top part of the eggshell. A square window opening (∼2 × 2 cm) was cut in each egg and covered with a transparent low adhesion tape. The eggs were incubated for an additional day. On the fifth day, sterilised samples of the inner and outer sides of the each membrane type (3 × 3 mm in size) were placed on the CAM. For controls, pre-sterilised with 70% ethanol, 3 × 3 mm Whatman #1 fiter paper squares, 20 µL PBS and 10 ng/ml vascular endothethial growth factor (VEGF)-loaded samples were used. All samples were carefully placed on the CAM, under sterile conditons, before the windows of the eggs were sealed. Eggs were then kept in the incuator for additional five days and monitored on a daily basis. On the 10th day, the tape/seal were removed and images taken using GX CAM digitial camera at ×1 magnification. Blood vessels were quantified, assessed and characterised using the AngioQuant software (MATLAB, UK).^42^

### Statistical analysis

All data were statistically analysed using PRISM (GraphPad software, version 9). The data were evaluated by analysis of variance (ANOVA) combined with Bonferroni’s Multiple post-test and Dunnett's Multiple Comparison Test (p < 0.05). Data presented were expressed as mean and standard deviation (SD).

## Results and discussion

### Thickness

The measured thicknesses of the extracted membranes are shown in Table 1. From the results, the thickness of the manually peeled membrane (ESM-strip) has an average thickness of 0.096 ± 0.005 mm, acetic acid (ESM-A0.5) has an average of 0.124 ± 0.01 mm and the thickness of EDTA extracted membrane (ESM-E0.9) has an average of 0.122 ± 0.014 mm. The results document that the thickness of the ESM-strip is significantly less than that of the ESM-A0.5 and ESM-E0.9 (p < 0.001) samples, whereas no significant differences can be observed between the ESM-A0.5 and ESM-E0.9 (p > 0.05) samples. A simple explanation for is relies on the fact the ESM consists of three distinct layers: the limiting membrane, inner shell membrane and the outer shell membrane. The manually peeled membrane results in the isolation of a membrane composing of two layers; the limiting membrane and the inner shell membrane. In contrast, owing to the fact that the outer shell membrane is firmly attached to the ESM and this layer can only be obtained by chemically treating the shell thereby releasing the membrane after the dissolution of the CaCO_3_.^14,18^ Previous studies have reported on the thicknesses of the three layers and have shown that each layer has a different and distinct thickness.^14,21^ Liang et al., using confocal scanning laser microscopy, reported that the average thicknesses of the limiting membrane, inner shell membrane and the outer shell membrane were 0.0036, 0.021 and 0.059 mm.^55^ In addition, other thicknesses have been reported in literature depending on how the membranes were prepared and the egg varieties assessed.^21,36^ Strnková et al.^39^ measured the thickness of manually peeled ESM from hen, goose and Japanese quails by using a digital micrometer and obtained ranges of 0.022–0.170, 0.033–0.110 and 0.040–0.090 mm, respectively, in their study. As such, these results obtained here are consistent with prior studies from existing literature.

**Table 1.** Thickness measurements of extracted membranes.

	Thickness (mm)		
	Minimum	Maximum	Mean ± SD
ESMstrip	0.080	0.110	0.096 ± 0.005
ESM-A0.5	0.100	0.150	0.124 ± 0.010^a^
ESM-A0.9	0.090	0.170	0.122 ± 0.014^a^

All values are expressed as mean ± SD for *n* = 6. One way ANOVA with Bonferroni’s Multiple Comparison post Test (p < 0.05), the average thickness of ESM-A0.5 and ESM-E0.9 are significantly higher than the ESMstrip.

^a^p < 0.001.

### Transparency

**Figure 4.**
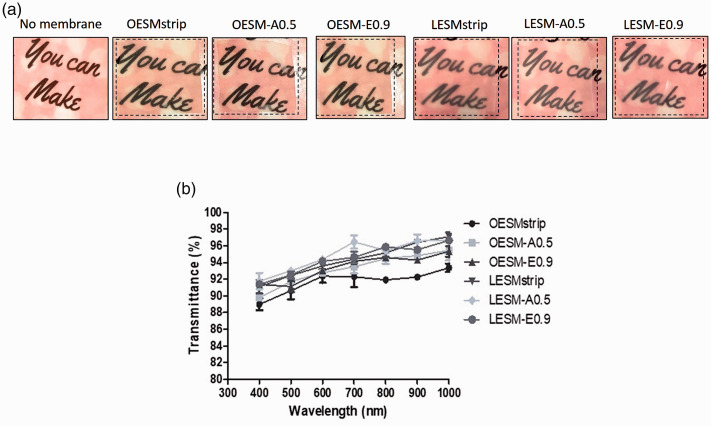
(a) Images representing the visual transparency of the outer and inner sides of the ESM samples (b) UV light transmittance profiles of membranes outer sides (OESMstrip, OESM-A0.5 and OESM-E0.9) and the inner side (LESMstrip, LESM-A0.5 and LESM-E0.9). O: outer side membrane; L: limiting (inner) membrane.

The cornea is the transparent window which plays a major role in the visual pathway and, accordingly, any material placed on it must be transparent in order not to compomise its principal role and function.^42,43^ Furthermore, during the application of dressing for wound healing, the transparency of the material is crucial for the visual observations of the wound healing process.^44^ The results of the visual observation of the inner and outer sides of each extracted membrane can be seen in Figure 4(a). The sample text can be clearly seen in the visual images from the inner side samples (LESM-strip, LESM-A0.5 and LESM-E0.9). In the case of the outer sides membrane samples, the visibility of the text in the OESM-strip, OESM-A0.5 and OESM-E0.9 are reduced although they can still be considered to be visually transparent overall. This concurs with results produced by Bellairs and Boyde ^21^ that the outer side of ESM is rough while the inner side is smooth which would directly impact visual clarity. Moreover, transparency of both sides of the membranes were further assessed by measuirng light transmittance using UV-VIS spectrophotometer and this data is summarised in Figure 4(b). Essentially, light transmittance values for LESM-strip, LESM-A0.5, LESM-E0.9, OESM-strip, OESM-A0.5 and OESM-E0.9 in the visible light (400–700 nm wavelengths) are all above 80%.

### Morphology

**Figure 5.**
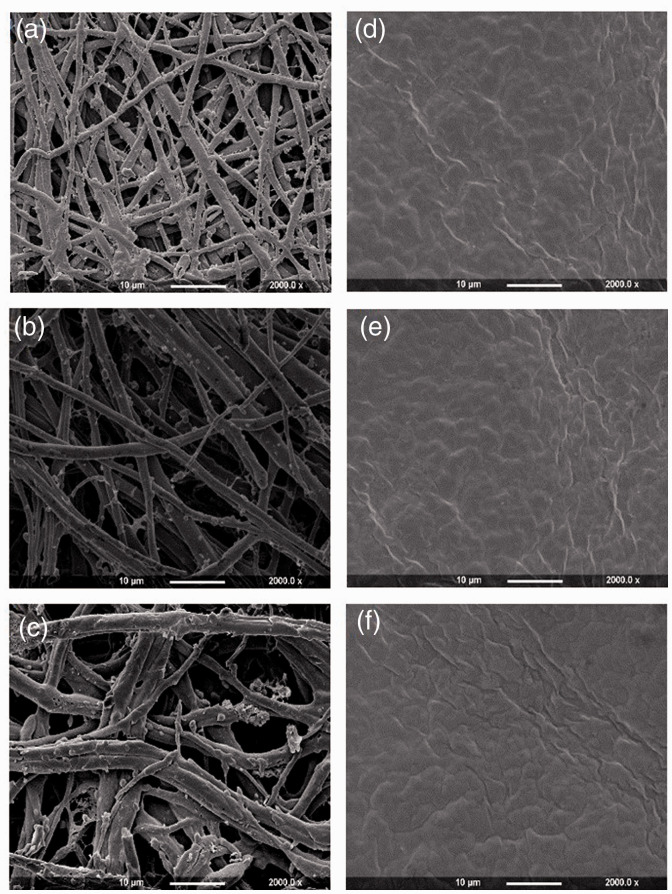
Field emission scanning electron microscopy (FESEM) images of the extracted ESM samples (a) OESMstrip, (b) OESM-A0.5, (c) OESM-A0.9, (d) LESMstrip, (e) LESM-A0.5 and (f) LESM-A0.9. O: outer side membrane; L: limiting (inner) membrane. Magnification at ×2000.

The outer and inner sides of extracted membranes were visualised by using FESEM and collated images can be seen in Figure 5. It can be noted that both sides of the ESM samples show different structural characteristics: the outer sides of the membranes (a, b and c) contain macroporous structures within networks of interwoven fibres. In contrast, inner side of the membranes, LESM-strip, LESM-A0.5 and LESM-E0.9 (Figure 5(d) to (f)) display a continuous dense structure with no other significant differences. On further analysis, the outer side of the OESM-strip (Figure 5(a)) which represents the inner shell membrane, displays similar fibrous structures to the OESM-A0.5 (Figure 5(b)) and OESM-E0.9 (Figure 5(c)) samples which are located as the outer shell. In comparison, the fibres of inner shell membrane appear to be much thinner than the outer shell membrane. Interestingly, these results are in conformity with previous reports in the literature.^18,21,45^

### Elemental/chemical composition

EDS analyses was used to determine the elemental composition of the membranes in order to profile remaining residues following the extraction process. From the results (Table 2), it can be seen that the ESM membranes compose of carbon, nitrogen, oxygen and sulphur. This corroborates the data stated by Tsai and colleagues that natural ESM ais made up of the elements C, N, O and S.^46^ Taking this further, EDS analysis of the individual inner and outer sides of the extracted membranes report the absence of the calcium content and is most likely due to the complete dissolution of the CaCO_3_ by the solvent treatment (i.e. acetic acid or EDTA). In short, these results demonstrate that both sides of the ESM contain the same bulk elemental compostion and that there is no stratified variation based on the membrane thickness/distinct layering.

**Table 2.** EDS spectra of (a) OESMstrip, (b) OESM-A0.5, (c) OESM-A0.9 of (d) LESMstrip, (e) LESM-A0.5, and (f) LESM-A0.9.

	Weight (%)					Atomic (%)				
	Carbon	Nitrogen	Oxygen	Sulphur	Calcium	Carbon	Nitrogen	Oxygen	Sulphur	Calcium
OESMstrip	40.70 ± 3.11	37.85 ± 4.73	18.24 ± 4.94	3.22 ± 1.66	0.00	46.30 ± 3.55	36.83 ± 4.57	15.54 ± 4.22	1.33 ± 0.71	0.00
OESM-A0.5	37.61 ± 3.91	39.13 ± 2.13	22.07 ± 3.42	1.16 ± 1.06	0.00	42.68 ± 4.22	38.05 ± 2.17	18.78 ± 2.97	0.49 ± 0.45	0.00
OESM-A0.9	39.72 ± 3.35	38.00 ± 2.96	21.01 ± 2.23	1.28 ± 0.70	0.00	44.82 ± 4.09	36.70 ± 3.10	17.92 ± 7.92	0.56 ± 1.65	0.00
LESMstrip	37.57 ± 4.23	40.58 ± 4.76	19.86 ± 1.98	1.98 ± 1.12	0.00	42.68 ± 2.77	39.53 ± 3.48	16.65 ± 3.17	0.84 ± 0.48	0.00
LESM-A0.5	38.84 ± 3.75	37.96 ± 3.14	21.98 ± 3.83	1.21 ± 0.77	0.00	41.46 ± 1.34	39.31 ± 2.09	18.77 ± 1.22	0.46 ± 0.46	0.00
LESM-A0.9	40.39 ± 4.12	40.17 ± 2.87	17.54 ± 2.50	1.93 ± 1.98	0.00	45.44 ± 3.82	38.92 ± 2.98	14.91 ± 2.19	0.73 ± 0.56	0.00

**Figure 6.**
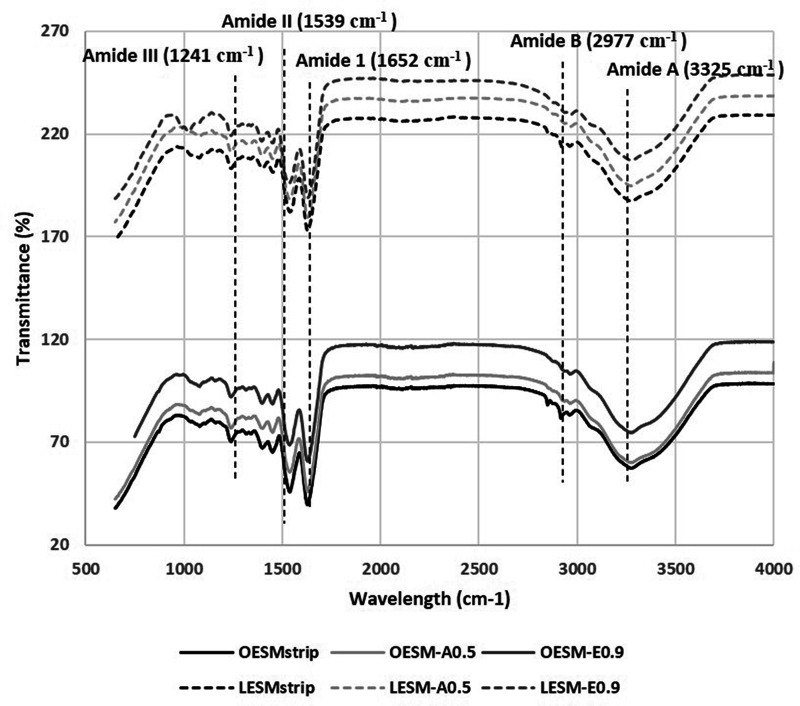
FTIR spectra summarising the chemical bonding structure of the extracted ESM samples. O: outer side membrane; L: limiting (inner) membrane.

FTIR spectroscopy was implemented to characterise the extracted membranes. Referring to Figure 6, the spectra of the inner and outer sides of each of the membranes are similar. The characteristic bands associated with the structural unit of proteins are identified in each spectrum.^14,19^ This supports the evidence in the literature that the fibres of ESM are mainly made up of proteins.^13–21^ The amide A band, identified at 3325 cm^−1^, corresponds to the N–H stretching and O–H groups, however the strong absorption of water has overlapped the band. The peak resolved at 2977 cm^−1^ represents amide B which is mainly associated the stretching vibration of C–H bonds found in =C–H and =CH functional groups.^47,48^ The peak at 1652 cm^−1^ (C = O) is associated to amide I band, 1530 cm^−1^, which corresponds to C≡N stretching, and N-H can be assigned to amide II band and the peak evidenced at 1241 cm^−1^.^19–21,43^ Baláž reported that these three lower peaks of the ESM spectrum correspond to the amide I, II and III vibration of the glycoproteins found in the fibres of the ESM.^20^ As such, the FTIR profiles suggest that the acetic acid and the EDTA mextraction protocols did not alter the chemical composition of the (organic) structure of the ESM.

### Fluid handling properties

The porosity of a material is vital in the cornea wound healing process as it influences other characteristics of the biomaterials such as moisture retention, permeability and strength.^2,4,7^ In Table 3, the membrane obtained manually, ESM-strip, has the lowest porosity profile of 56.54%. In contrast, membranes extracted by EDTA, ESM-E0.9, reported to have the highest porosity measurment of 69.38%. This observation might be ascribed to the fact that the pore size of the outer side of the ESM-strip, the inner shell membrane, is lower than that of the outer side of ESM-A0.5 and ESM-E0.9, outer shell membrane as reported previously.^31,45,49^ However, despite these observations, there are no significant difference among the three types of extracted membranes (p = 0.2540) when compared directly.

**Table 3.** Porosity, fluid adsorption and weight loss% of different extracted membranes.

		Fluid adsorption (%)		
	Porosity (%)	1 h	24 h	Weight loss (%)
ESMstrip	56.54 ± 8.26	232.24 ± 33.23	229.97 ± 27.53	72.58
ESM-A0.5	63.06 ± 11.42	291.49 ± 34.50	284.88 ± 20.53	58.71
ESM-A0.9	69.38 ± 3.85	317.48 ± 29.28	335.45 ± 34.77	51.47

The fluid absorption capacity of the extracted membranes was assessed at the 1 and 24-h timepoint. From the results (Table 3), no significant differences were observed on comparing the two timepoints between the ESM-strip, ESM-A0.5 and ESM-E0.9 samples. However, the fluid absorption capacity of the ESM-strip was noticeably lower than that of the ESM-A0.5 sample (p < 0.01). A similar trend was also observed in the comparison between the ESM-strip and ESM-E0.9 samples. There was no significant difference between the fluid absorption capacities of ESM-A0.5 and ESM-E0.9 (p > 0.05).

### Thermal properties

The thermal behaviour of the extracted membranes was examined by TGA experiments. The percentage weight loss for each type of membrane was deduced from the TGA curve and the results are summarised in Table 3. Overall, the % weight loss of ESM-strip was higher (72.58%) than that of the ESM-A0.5 (58.71%) and the ESM-E0.9 (51.47%) sample. It has been reported that differences in the thermal stabilities of the membranes are dependent on the structure and chemical composition^50–52^ and, as such, the obtained data corroborates the results obtained for the fluid handling properties above.

### Wettability

**Figure 7.**
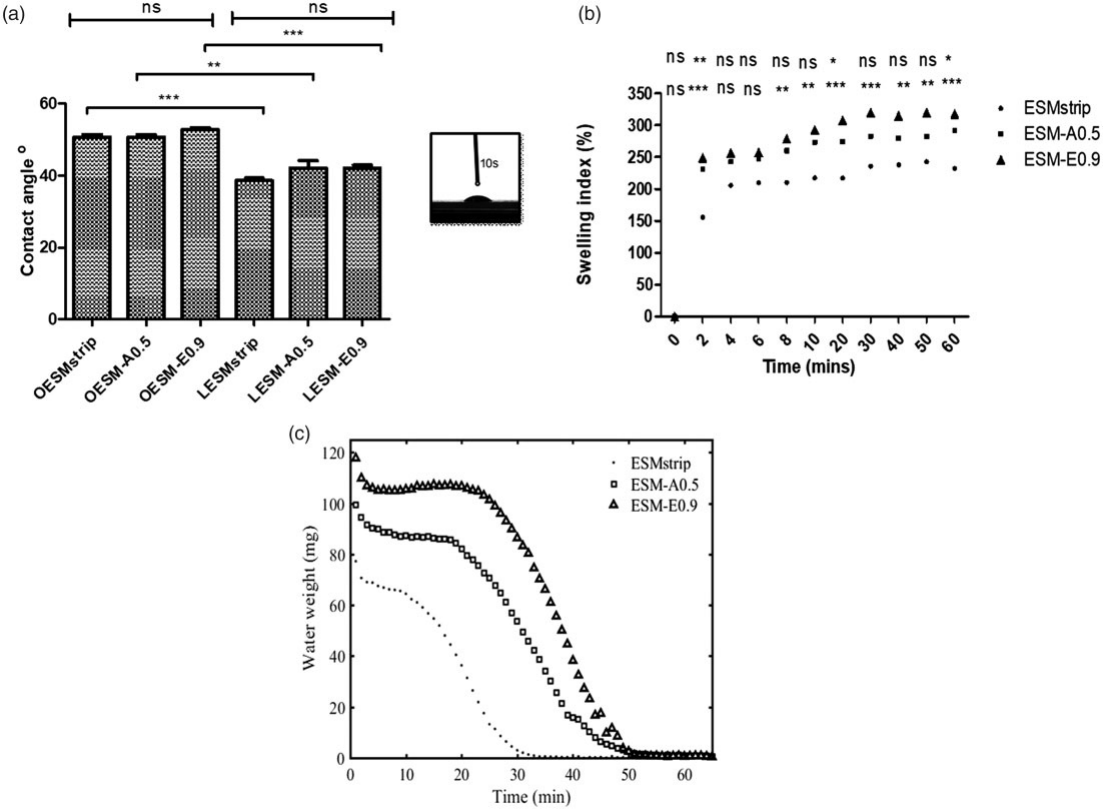
Water characterisation of ESM samples: (a) water contact angles of extracted membranes. One-way ANOVA with Bonferroni’s Multiple Comparison post Test (p < 0.05). All values are expressed as mean ± SD for *n* = 6. (**p < 0.01; ***p < 0.001; ns: no significant difference); (b) Swelling profiles of the extracted membranes. Two-way ANOVA with Bonferroni’s Multiple Comparison post Test (p < 0.05). All values are expressed as mean ± SD for *n* = 6. (*p < 0.05; **p < 0.01; ***p < 0.001; ns: no significant difference); (c) desorption profiles for ESMstrip, ESM-A0.5 and ESM-E0.9.

Contact angle measurements for the ESM samples are can be seen in Figure 7(a). Noticeably, significant differences between the contact angle values of the inner side of the membranes (OESM-strip, OESM-A0.5 and OESM-E0.9) and the outer side of the membranes (LESM-strip: p < 0.001, LESM-A0.5: p < 0.01 and LESM-E0.9: p < 0.001) were observed. These values demonstrate the hydrophilicity of the extracted membrane and this characteristic could contribute to the adhesion and spreading of cells on the membrane surface as previously suggested.^53^ It is assumed that the low hydrophilicity of the outer side of the membrane is attributed to its interwoven fibrous structure and rough surface. In contrast, the relatively smooth surface and dense structure of the inner side, the limiting membrane, may contribute to its high hydrophilic behaviour. Above all, no significant difference among the respective inner and outer sides of the extracted membrane (p > 0.05) were observed and these results can be seen to be in a good agreement with previous published studies.^48,53–55^

Swelling profile is one of the important characteristics that determine fluid retention, erosion and hydrophilic of a material with potential application for an ocular wound dressing.^7,51,56^ In this study, the swelling profiles of ESM-strip, ESM-A0.5 and ESM-E0.9 were assessed and the results summarised in Figure 7(b). In the first 2 min, all three membranes swelled rapidly, however the swelling capacities of the ESM-A0.5 and ESM-E0.9 were significantly higher (p = 0.01 and p < 0.001, respectively) than that of the ESM-strip sample. After 10 min, the swelling capacities of all the extracted membranes remained relatively constant. It can be noted that the swelling prolife of the ESM-strip was significantly lower than the ESM-A0.5 (p < 0.05) and ESM-E0.9 (p < 0.001) samples. In contrast, no significant difference was observed between the ESM-A0.5 and the ESM-E0.9 (p > 0.05) samples.

### Water drying profile

The membrane water desorption profiles for ESMstrip, ESM-A0.5 and ESM-E0.9, are shown in Figure 7(c), which qualitatively shows a similar trend to the swelling profiles, with ESM-E0.9 having the highest swelling due to fluid retention, followed by ESM-A0.5 and ESMstrip. It is also interesting to note that at a decreasing time, a similar pattern can be observed i.e. a period of constant water weight followed by a steady decrease, which is consistent with the WCA data. The sharp decline is also consistent with the logarithmic decay behaviour observed in polymer based membranes.^34,57^ The sharp weight loss in the first 3 min is possibly due to evaporation of the excess surface water.

### Tensile properties

**Figure 8.**
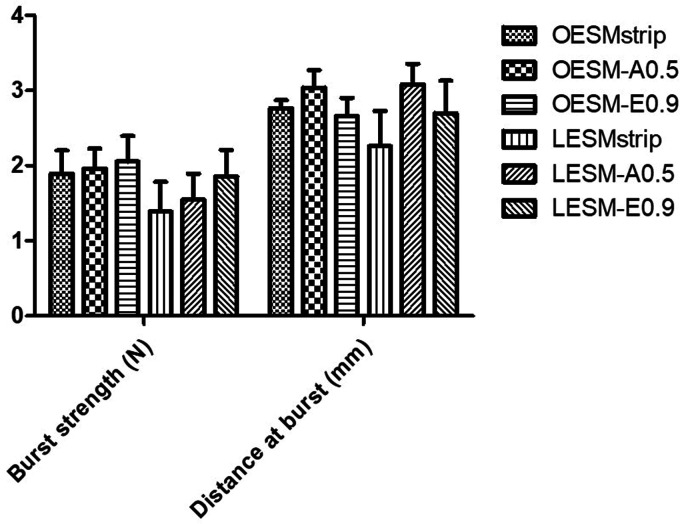
Compression profile (burst strength and distance at burst) for OESMstrip; OESM-A0.5; OESM-A0.9 of LESMstrip; LESM-A0.5; and LESM-A0.9.

The mechanical behaviours of potential biomaterials are crucial to their performance.^25^ The uniaxial tension and uniaxial compression were employed for the measurement of tensile strength/Young’s modulus/percentage elongation at break and burst strength of the fabricated membranes, respectively. The stiffness of the inner and outer sides of each membrane were evaluated and compared in context of the compression properties. The burst strength, a measure of resistance to rapture was recorded for the inner and outer sides of each membrane, and is dependent on the tensile strength, and the porosity of the material.^33^ Taken together, the results obtained for the burst strength and distance to burst of both the inner (LESM-strip, LESM-A0.5 and LESM-E0.9) and outer sides (OESM-strip, OESM-A0.5, OESM-E0.9) of each extracted membranes are illustrated in Figure 8 and Table 4. On comparison, OESM-strip (1.89 N), OESM-A0.5 (1.96 N), OESM-E0.9 (2.06) require high forces to break, and demonstrate slight expandable characteristics; the LESM-strip (2.26 N), LESM-A0.5 (1.55 N) and LESM-E0.9 (1.86 N) need lower forces to rupture accordingly. Primarily, the components and interactions in the respective side of the ESM samples dominate the strength to burst and may be related to their structural characteristics- as previously stated, the outer side of ESM is composed of fibres whereas the inner side has significantly less.^14,15,20^ However, no significant differences (p > 0.05) were observed when comparing the OESM-strip, OESM-A0.5 and OESM-E0.9. In respect of the inner sides of the membranes, the differences between LESM-strip/LESM-A0.5 and LESM-A0.5/LESM-E0.9 (p > 0.05) were determined not to be signifcant whereas a signifcant difference was obtained between the LESM-strip/LESM-E0.9 (p < 0.001) samples.

**Table 4.** Results of two-way ANOVA with Bonferroni’s multiple comparison post test (p < 0.05).

Sample	Burst strength (*N*)	Distance (mm)
OESMstrip vs OESM-A0.5	ns	ns
OESMstrip vs OESM-E0.9	ns	ns
OESMstrip vs LESMstrip	*	*
OESMstrip vs LESM-A0.5	ns	ns
OESMstrip vs LESM-E0.9	ns	ns
OESM-A0.5 vs OESM-E0.9	ns	ns
OESM-A0.5 vs LESM-A0.5	*	***
OESM-A0.5 vs LESM-E0.9	ns	ns
OESM-E0.9 vs LESMstrip	**	ns
OESM-E0.9 vs LESM-A0.5	*	ns
OESM-E0.9 vs LESM-E0.9	ns	ns
LESMstrip vs LESM-A0.5	ns	***
LESMstrip vs LESM-E0.9	*	ns
LESM-A0.5 VS LESM-E0.9	ns	ns
OESMstrip vs OESM-A0.5	ns	ns
OESMstrip vs OESM-E0.9	ns	ns
OESMstrip vs LESMstrip	*	*

All values are expressed as mean ± SD for *n* = 6. (*p < 0.05, **p < 0.01 ***p < 0.001; ns: no significant difference).

The results of the uniaxial tension tests of the extracted membranes are listed in Table 5 indicating no significant differences between the ultimate tensile strength and Young’s modulus of the manually detached membrane, ESM-strip and the chemically treatment method; ESM-A0.5 and ESM-E-0.9 (p > 0.05) were observed. As tensile strength is defined as the maximum stress that a material can withstand while being stretched, it can often be associated with (or defined) as the toughness/strength of a material. In contrast, the measure of stiffness is derived from the Young’s modulus value.^33^ From the data, significant differences between the percentage elongation at break of the ESM-strip and ESM-A0.5 (p < 0.001) and ESM-strip and ESM-E0.9 (p < 0.01) samples were observed. The decrease in thickness of the ESM-strip contributed to the lower % elongation at break. No significant difference was observed between the ESM-A0.5 and ESM-E0.9 (p > 0.05) sample. As such, it can be proposed that the mechanical behaviour of ESM is influenced by the distortion of the (alignment) proteins within the fibres of the membrane.^33^ The values obtained for these ESM membranes seemingly contribute to the evidence reported in literature that ESM are tough and stiff materials.^25,31,39^

**Table 5.** Tension profile of extracted membrane (mean ± SD, *n* = 6).

	Ultimate tensile strength (MPa)	Elongation (%)	Young’s modulus (MPa)
ESMstrip	0.945 ± 0.272 (ns)	22.567 ± 5.131 (***)	4.164 ± 0.422 (ns)
ESM-A0.5	1.340 ± 0.163 (ns)	40.453 ± 5.270 (***)	3.322 ± 0.213 (ns)
ESM-A0.9	1.442 ± 0.262 (ns)	37.974 ± 3.436 (**)	3.647 ± 0.235 (ns)

Statistics was performed using a two-way ANOVA with Bonferroni’s Multiple Comparison post Test (p < 0.05), All values are expressed as mean ± SD for *n* = 6. ((**p < 0.01 ***p < 0.001; ns: no significant difference).

### In vitro cytotoxicity

**Figure 9.**
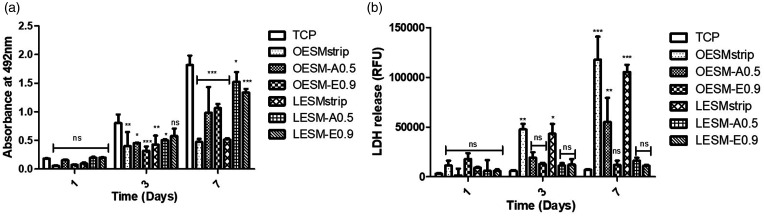
Cell metabolic activity and LDH release of iHCE cells cultured on tissue culture plate, inner and outer sides of extracted membranes at 10^4^ cells per well density over seven days. Data are represented as mean ± SD (*n* = 3) with statistical assessment performed by using the two-way ANOVA with Bonferroni’s Multiple Comparison post Test (p < 0.05). *corresponds to p < 0.05; **corresponds to p < 0.01; *** corresponds to p < 0.001; ns: no significant difference: (a) comparison of iHCE cell metabolic activity cultured on different membranes/surfaces; (b) comparison of iHCE LDH release when cultured on different membranes/surfaces.

**Figure 10.**
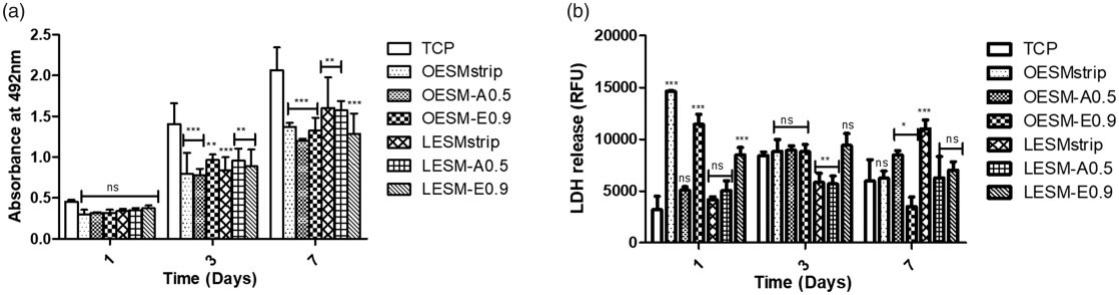
Cell metabolic activity and LDH release of C-MSC cells cultured on tissue culture plate, inner and outer sides of extracted membranes at 10^4^ cells per well density over seven days. Data are represented as mean ± SD (*n* = 3) with statistical assessment performed by using the two-way ANOVA with Bonferroni’s Multiple Comparison post Test (p < 0.05). * corresponds to p < 0.05; ** corresponds to p < 0.01; *** corresponds to p < 0.001; ns: no significant difference: (a) comparison of C-MSC cell metabolic activity cultured on different membranes/surfaces; (b) comparison of C-MSC LDH release when cultured on different membranes/surfaces.

Mitochondrial activity and cell death of the iHCE and C-MSC cells were measured using the MTS and LDH assays, respectively, following *in vitro* culture on the different ESM samples for up to seven days. As can be seen in Figures 9 and 10, a noteable effect of the inner sides of ESM samples (i.e. LESM-strip, LESM-A0.5 and LESM-E0.9) on the metabolic activities of both cell lines were observed than during culture on the outer side membrane samples (i.e. OESM-strip, OESM-A0.5, OESM-E0.9). In the context of the iHCE cells, after 24 h of culture, no significant difference was observed in each membrane type as compared to the control (TCP). Moreover, on day 3 of culture, no significant differences was observed in cells cutlured on LESM-E0.0. However, signficant differences were recorded for LESM-strip (p < 0.001), LESM-A0.5 (p < 0.05), OESM-strip ((p < 0.05), OESM-A0.5 and OESM-E0.9. The metabolic activities of all the samples increased after day 3 except for the LESM-strip and OESM-strip samples. Lactate dehydrogenase (LDH) is released by cells following a compromised cell membrane i.e. cell death and, as such, the collated LDH results correlate to the mitochondrial activity/MTS data. Figure 10 compares the effect of the inner and outer side of the membranes on C-MSC cell function: in short, no statistically different observations could be made between the samples and the control following 24 h of cell incubation (p > 0.05). Intriguingly, a linear increase in the mitochodrial functions of cells attached to ESM were observed over the seven days and suggest that the cell may be growing in a non-exponential manner but, more importantly, not being subjected to apoptosis and/or necrotic cell death.

**Figure 11.**
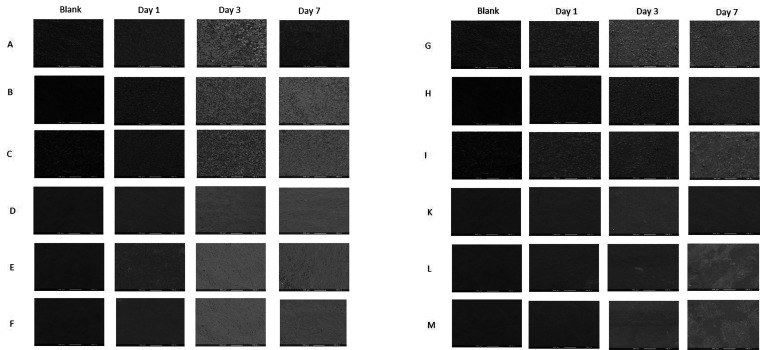
SEM images showing the attachment and spreading of iHCE cells on the different substrates: (a) ESMstrip, (b) OESM-A0.5, (C) OESM-E0-9, (D) LESMstrip, (E) LESM-A0.5 and (F) LESM-E0.9. SEM images showing the attachment and spreading of C-MSC cells on the different substrates (g), OESMstrip (h), OESM-A0.5 (i), OESM-E0-9 (j), LESMstrip (k) and LESM-A0.5 (l) LESM-E0.9. Cells were cultured for one, three and seven days. Magnification: ×100.

Figure 11 summarises the attachment and spreading of iHCE and C-MSC cells on OESM-strip, OESM-A0.5, OESM-E0.9, LESM-strip, LESM-A0.5 and LESM-E0.9 samples. It appears that due to the relatively increase in surface area (or smooth topography) of the surface of the inner side of ESM (i.e. LESM-strip, LESM-A0.5 and LESM-E0.9), a great number of cells adhered compared to the contrasting outer side membrane samples (i.e. OESM-strip, OESM-A0.5 and OESM-E0.9). This characteristic may seemingly corroborate the observed biocompatability and biological properties of the cells in terms of degree of attachment, spreading and proliferation of the cells on the ESMs.^43,58^

### Angiogenic response

**Figure 12.**
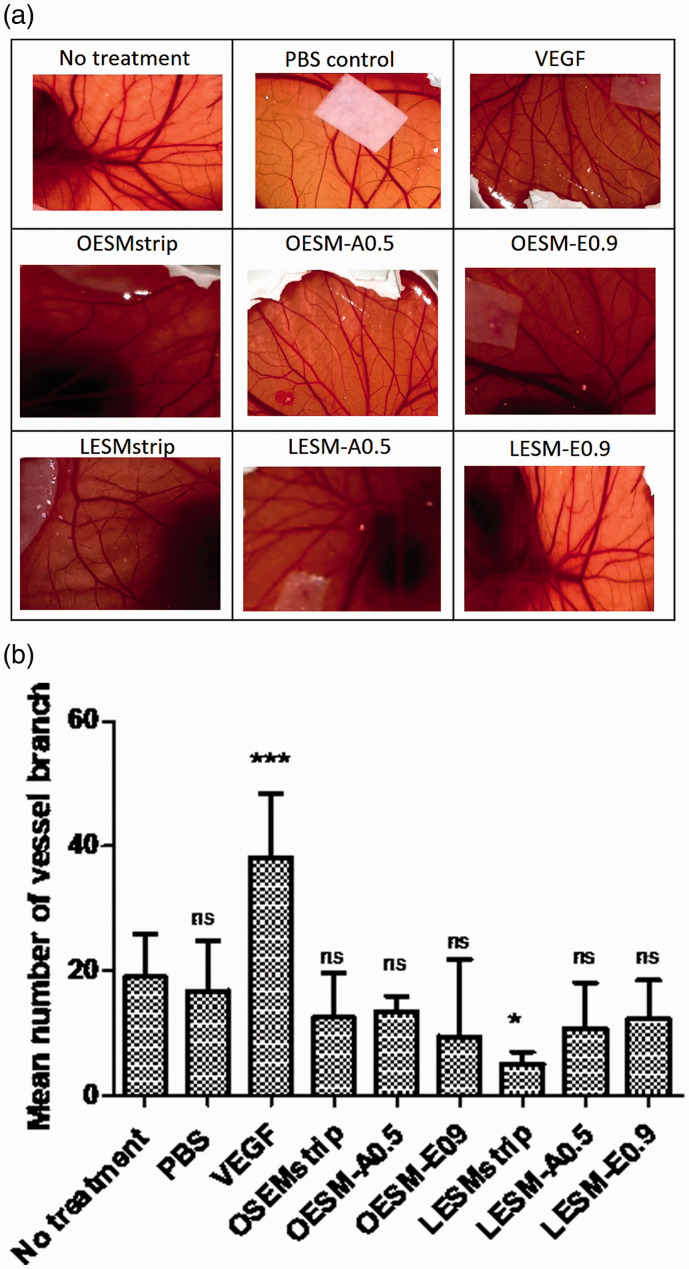
(a) Photographs of the CAM assays, after 10 days of incubation at 37°C, in the presence of extracted ESM samples and controls. VEGF; vascular endothelial growth factor. (b) Graph of number blood vessel branches measured using AngioQaunt software expressed as mean ± SD. * corresponds to p < 0.05; *** corresponds to p < 0.001; ns: no significant difference.

Angiogenesis plays a vital role in wound healing and the presence of pro-and anti-angiogenic factors can therefore influence the response and regulation of blood vessel formation.^59–62^ In addition, the physical structure and/or properties of a novel scaffold material may also influence the ability as well as the direction of vessel growth- as seen in the SEM images, the ESM has a degree of porosity within its innate structure. In order to evaluate the angiogenic profile of the ESM, the CAM assay was employed. Figure 12(a) and (b) summarise the results obtained from the CAM assay where no treatment, filter paper loaded PBS control, VEGF-a key mediator angiogenic agent,^62,63^ LESM-strip, LESM-A0.5, LESM-E0.9, OESM-strip, OESM-A0.5 and OESM-E0.9 were used and compared to each other. After 10 days of incubation within the CAM, samples were imaged and the number of branching vessels were determined using AngioQuant software. From the results (Figure 12(b)), the VEGF-loaded sample demonstrated the greatest number of vessel branches (p < 0.001), whereas the lowest number of vessels was seen with the inner side of manually peeled ESM, LESM-strip sample (p < 0.05). Intriguingly however, the PBS control, LESM-A0.5, LESM-E0.9, OESM-strip, OESM-A0.5 and OESM-E0.9 samples did not significantly increase angigenesis as compared to the no treatment CAM. No data has been reported on the angiogenic potential of ESM to date and, as such, further studies may need to be employed to confirm the behaviour and/or mechanism behind these results. However, it is importantly to note that no determental effect had been observed with the ESM samples within the CAM assay. In the context of a biomaterial, having pro- and/or angiogeneic capability may be considered as a positive or negative characteristics e.g. minimal blood vessels for optical applications (i.e. transparency) whereas increasesd blood vessels would be advantageous for a dermal wound dressing.

## Conclusion

In this study, we describe three successfully optimised protocols that can be used to extract the intact membrane from the eggshell without comprising its innate structure or physico-chemical characteristics. Accordingly, each specific protocol results in the isolation of an ESM that has defined properties which include membrane thickness, structural arrangement, porosity, swelling profiles, hydrophilicty, elemental composition and transparency. Biocompatibility of these ESM were also assessed using cell culture and demonstrated minimal adverse effects- in some instances, increasing cell attachment, spreading and proliferation of the cells. Taken together, these results demonstrate that the ESM could be exploited in a number of regenerative medical and/or biotechnological applications such as a wound dressing for ocular injury, given the high transparency of the biomaterial. The membrane also has potential as a culture substrate for the drug discovery pipeline. Such a material would also mitigate issues regarding ethics and tissue availaibilty as well as encouraging “green technology” of converting a low-cost waste material to a product of signifiantly higher value.
